# Cost-effectiveness analysis of atezolizumab plus bevacizumab versus sorafenib in first line treatment for Chinese subpopulation with unresectable hepatocellular carcinoma

**DOI:** 10.3389/fonc.2023.1264417

**Published:** 2023-11-08

**Authors:** Chien-Yu Tseng, Yi-Wen Tsai, Ming-Neng Shiu

**Affiliations:** ^1^ Department of Pharmacy, National Yang Ming Chiao Tung University, Taipei, Taiwan; ^2^ Institute of Health and Welfare Policy, National Yang Ming Chiao Tung University, Taipei, Taiwan

**Keywords:** atezolizumab plus bevacizumab, sorafenib, advanced hepatocellular carcinoma, cost-effectiveness analysis, partitioned survival model

## Abstract

**Objective:**

We aimed to evaluate the cost-effectiveness of atezolizumab plus bevacizumab (atezo-bev) versus sorafenib treatment in Taiwan.

**Methods:**

Using sorafenib as the comparator, we developed a partitioned survival model to evaluate the costs and quality-adjusted life year (QALY) of the atezo-bev treatment. The time horizon of the study was 15 years, and the annual discount rate was 3%. We analyzed the incremental cost-effectiveness ratio (ICER) and incremental net monetary benefit (INMB) from the treatment effects (determined from the progression-free and overall survival outcomes of the IMbrave150 study), direct medical costs (collected and estimated from the National Health Insurance Research Database, Taiwan), and utility parameters (referred to the NICE technology appraisal guidance), as well as the deterministic sensitivity and probabilistic sensitivity.

**Results:**

Compared with sorafenib, the incremental effectiveness of atezo-bev treatment was 1.7 QALY, with an incremental cost of USD 127,607. The ICER was USD 75,192 per QALY, which was less than the predefined willingness to pay in Taiwan.

**Conclusion:**

The combined treatment of atezo-bev is cost-effective when compared with sorafenib, which is currently the first-line treatment option for unresectable HCC in Taiwan.

## 1 Introduction

Among the most common cancers, liver cancer has the third highest mortality rate (1). Although liver cancer deaths are declining in Taiwan, it remains the second leading cause of cancer-related deaths (2). According to the latest Taiwan Cancer Registry report, 8,638 patients were diagnosed with hepatocellular carcinomas (HCC), which accounts for 90% of all liver cancers (3).

The Barcelona Clinic Liver Cancer (BCLC) system has widely standardized clinical staging of HCC (4), categorizing HCC into stages 0, A, B, C, and D. Patients with early-stage HCC are asymptomatic (5); therefore, more than 50% of patients are diagnosed with BCLC stage C (advanced HCC) (6) when tumor cells may invade the hepatic and/or portal veins, with limited treatment options and poor prognoses. Targeted therapies play an important role in the treatment of advanced HCC because they are more effective than chemotherapy. In Taiwan, multi-kinase inhibitors, such as sorafenib and lenvatinib, are used as first-line treatments for BCLC stage C HCC. Several types of biologics and immunotherapies have been developed in recent years to treat advanced HCC. The IMbrave150 study (7) in 2020 reported that atezolizumab plus bevacizumab (atezo-bev) can significantly improve patients’ overall survival time compared to sorafenib. From the IMbrave150 clinical trial, the hazard ratio (HR) for overall survival (OS) was 0.58 (95% CI, 0.42–0.79), and the hazard ratio for progression free survival (PFS) was 0.59 (95% CI, 0.47–0.76) (7). Furthermore, atezo-bev had fewer treatment-related adverse events than sorafenib (7). Therefore, atezo-bev was listed as the first-line treatment for unresectable HCC in the National Comprehensive Cancer Network (NCCN) Hepatobiliary Cancer version 1.2022 (8). This combination treatment was approved by the Taiwan Food and Drug Administration for treating unresectable or metastatic Child-Pugh class A HCC (9).

Although atezo-bev can prolong the survival time of patients with HCC, the treatment costs are more expensive than those of sorafenib. A 60 kg patient needs to pay USD 10,348 monthly (10), which is more expensive than other targeted therapies, such as sorafenib (USD 3,452/month) or lenvatinib (USD 3,642/month) in Taiwan (11). Previous studies from the United States, Canada, and the United Kingdom found that atezo-bev is not cost-effective in the treatment of unresectable hepatocellular carcinoma (12–17). The incremental cost-effectiveness ratio (ICER) ranged between USD 169,223 and USD 607,894 per quality-adjusted life-year (QALY) (12–17). These prior studies have primarily extracted efficacy data from the global population of IMbrave150 clinical trial results and have utilized corresponding local costs to assess the cost-effectiveness of atezo-bev compared to sorafenib. However, it is worth noting that HCC is particularly prevalent in the Asian region, and the IMbrave150 study demonstrated that atezo-bev treatment is associated with greater efficacy in the Chinese subpopulation, as evidenced by a stratified HR for OS of 0.44 (95% CI 0.25–0.76) compared to the global patient cohort. The stratified HR for PFS was 0.60 (95% Cl, 0.40–0.90), aligns closely with the global result (18). Despite this promising observation, it is noteworthy that there has been a conspicuous absence of cost-effectiveness analyses that specifically target the Asian or Chinese subpopulation.

As the hazard ratio was not significant between the Chinese subpopulation and the total population, we were uncertain whether it would affect the cost-effectiveness. Therefore, we cannot determine whether atezo-bev is cost-effective based on the efficacy from Chinese subpopulation and existing cost and in Taiwan. Since the 2-year survival rate of BCLC stage C HCC is only 17.8% (19), patients with HCC still need new treatments to prolong their survival time. Thus, this study aimed to compare the cost-effectiveness of atezo-bev and sorafenib as the first-line treatment for unresectable hepatocellular carcinoma (HCC) from the payer perspective of Taiwan’s National Health Insurance Administration (NHIA).

## 2 Methods

### 2.1 Decision model overview

**Figure 1:**
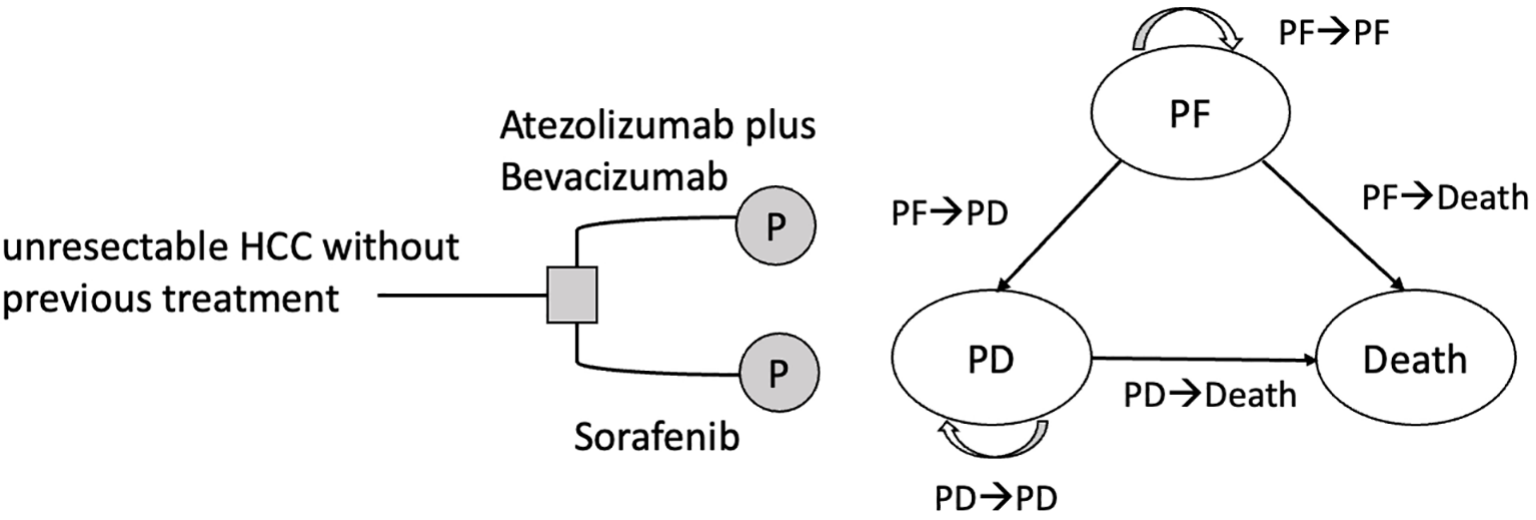
Partitioned survival model for unresectable HCC with three states. HCC, hepatocellular carcinoma; PF, progression-free; PD, progressive disease.

Similar to the IMbrave150 trial (18), the study population included patients with locally advanced metastatic and/or unresectable HCC who had not received prior systemic therapy. Those with a history of autoimmune diseases, HBV and HCV coinfections, bleeding esophageal conditions, or malignancies within the previous 5 years were excluded from the study. According to the treatment regimens used in the IMbrave150 trial (18), patients were administered either an atezo-bev regimen or sorafenib. Atezolizumab was administered intravenously at a dosage of 1,200 mg every 3 weeks, concomitant with bevacizumab at a dose of 15 mg/kg. Alternatively, patients receiving sorafenib were orally administered a dosage of 400 mg twice daily. To analyze the cost-effectiveness of atezo-bev and sorafenib, we constructed a partitioned survival model (PartSM) and divided survival time into three states: progression-free (PF), progressive disease (PD), and death (**Figure 1**). If the patient was receiving first-line treatment and had not developed any disease progression, they were considered to be in the PF state. PD was defined as a disease stage in which either the need for second-line treatment, regardless of disease progression or intolerance of adverse effects, or best supportive care was required. Since the 7-year overall survival rate for BCLC stage C HCC patients in Taiwan is 6.8% (20), the study time horizon was set as 15 years to assess patients’ life expectancy. The cycle length was set to 1 month according to the treatment regimen of atezo-bev and sorafenib (16). Based on the payer perspective of the NHIA, we collected the treatment effects of atezo-bev and sorafenib from progression-free survival (PFS) and overall survival (OS) curves in the IMbrave150 trial (Chinese subpopulation) (18), direct medical costs from the National Health Insurance Research Database (21, 22), and utility parameters from the NICE technology appraisal guidance (23).

### 2.2 Treatment effects

We extracted PFS and OS Kaplan-Meier curves from the Chinese subpopulation of the IMbrave150 trial to reconstruct individual patient data (IPD) of patients with unresectable or metastatic HCC without prior systemic treatment and extrapolated the survival curve beyond the limited follow-up time to determine the treatment effects of atezo-bev and sorafenib. Overall, 194 patients were enrolled in the study, including 59 from Taiwan and Hong Kong and 135 from mainland China (18). Based on Guyot et al. (24), we digitized the survival curves from the Chinese subpopulation of the IMbrave150 trial to generate individual patient data using WebPlotDigitizer, version 4.4 (25).

We selected the most fitted survival model from the various types of parametric survival models with Akaike information criterion (AIC) and visual inspection and used external real-world data and expert opinions to validate the selected model. According to AIC, the preferred model was log-logistic model. However, the appropriateness of our model survival curve was justified by the statistics from Taiwan Cancer Registry, which show that overall of 3-, 5-, and 7-year survival rates for BCLC stage C HCC patients were 14.6%, 9.6%, and 6.8%, respectively (20). Therefore, by using parametric survival model, we chose lognormal model to extrapolate the survival curve beyond the limited follow-up period. Finally, we calculated life expectancy by plotting PFS and OS curves for different treatment strategies and evaluated the area under the curve of the PFS and OS curves of the partitioned survival model.

### 2.3 Treatment costs and utility

From the payer perspective of the NHIA, we estimated the direct medical costs of treating advanced/metastatic or unresectable HCC using the National Health Insurance Research Database (NHIRD) from the Health and Welfare Data Science Center, Ministry of Health and Welfare (HWDC, MOHW). It is the largest medical expenditure database in Taiwan, derived from claims data, and contains complete medical expense records for over 99% of the population (21, 22). The NHIRD provides information on demographics, diagnoses, procedures, prescriptions, and expenditures under this single-payer national health insurance program (21). In order to access NHIRD, we obtained Institutional Review Board (IRB) approval from National Yang Ming Chiao Tung University (IRB No. YM110172E).

In this study, we included datasets such as ambulatory care expenditures by outpatient visits (CD), details of ambulatory care orders (OO), inpatient expenditures by admissions (DD), details of inpatient orders (DO), and cause-of-death data (26). Direct medical costs were determined by analyzing these datasets, including the costs in the PF and PD states. Costs in the PF state include medication, standards of care, and adverse event (AE) costs. To calculate the monthly medication cost of atezo-bev, we assumed that the patient weighed 60 kg. According to the NHI reimbursement fees for atezolizumab and bevacizumab for treating other cancers, the medication cost of atezo-bev was estimated to be USD 10,066/month. Sorafenib medication cost was USD 3,452/month for the treatment of patients with HCC. The average standard of care cost was USD 1,017 for treating HCC. In the atezo-bev group, the monthly costs for adverse events were USD 31, whereas they were USD 13 per month for the sorafenib group. Since there was no clinical information in the NHIRD about time to progression, we defined the date of progression as the last prescription date of first-line treatment plus the number of total days of drug use. Costs in the PD state included second-line treatment and best supportive care costs, which were USD1,443/month, and end-of-life care costs, which were USD 1,996 and USD 2,218 in the atezo-bev and sorafenib groups, respectively.

The study also considered the utility of evaluating quality-adjusted life years. Because of the lack of published Taiwanese utility data, utility values were determined using the NICE technology appraisal guidance derived from EQ-5D data collected during the IMbrave150 study. The utility of the atezo-bev group was 0.74 at pre-progression, including AE disutility, whereas that of the sorafenib group was 0.72. The utility value was 0.72 after entering the post-progression state and accounting for AE disutility (23).

### 2.4 Base case analysis

We compared atezo-bev against sorafenib to analyze incremental cost-effectiveness ratio (ICER), incremental net monetary benefit (INMB) from the treatment effects, direct medical costs and utility parameters (**Table 1**). The value of ICER and INMB was based on the following equations: ICER= (Catezo-bev−Csorafenib)/(Eatezo-bev −Esorafenib) = ∆C/∆E and INMB =λ ×∆E –∆C. If the ICER was lower than the willingness to pay (λ) and INMB was more significant than 0, atezo-bev would be a cost-effective treatment strategy, and vice versa. The study determined an annual discount rate of 3% due to Taiwan’s economic growth and interest rates (27). The willingness to pay (WTP) threshold was determined as three times per capita gross domestic product (USD 92,480 per additional QALY gained) (28) in Taiwan.

**Table 1:** Model inputs parameters.

Variable	Baseline	Range	Distribution	Ref.
Parameters of survival				
Atezolizumab plus bevacizumab (atezo-bev)				
**PFS of atezo-bev**	meanlog=1.796 sdlog=1.132	meanlog (1.573, 2.020) sdlog (0.951, 1.346)	lognormal	(18)
**OS of atezo-bev**	meanlog=3.370 sdlog=1.467	meanlog (2.780, 3.960) sdlog (1.075, 2.001)	lognormal	(18)
Sorafenib				
**PFS of sorafenib**	meanlog= 1.216 sdlog= 0.955	meanlog (0.943, 1.489) sdlog (0.759, 1.203)	lognormal	(18)
**OS of sorafenib**	meanlog=2.309 sdlog=1.129	meanlog (1.918, 2.699) sdlog (0.838, 1.521)	lognormal	(18)
Parameters of costs (USD per patient, month)				
Medication/month				
**1) AE of atezo-bev**	USD 10,066	Fixed	Gamma	NHIRD
**2) AE of sorafenib**	USD 3,452	Fixed	Gamma	NHIRD
**Standard of care (PF)/month**	USD 1,017	(63.3, 3,663.3)	Gamma	NHIRD
Adverse events (AE)/month				
**1) EoLC of atezo-bev**	USD 31	(13.3, 55.5)	Gamma	NHIRD
**2) EoLC of sorafenib**	USD 13	(6.6, 22.4)	Gamma	NHIRD
**Progressive disease/month**	USD 1,443	(54.6, 4316.8)	Gamma	NHIRD
**atezo-bev_ EoLC**	USD 1,996	(114.1, 7039)	Gamma	NHIRD
**Sorafenib_ EoLC**	USD 2,218	(126.8, 7821.1)	Gamma	NHIRD
Parameters of utility (per patient, month)				
**PFS of atezo-bev**	0.74	(0.728, 0.764)	Beta	(23)
**PFS of sorafenib**	0.72	(0.695, 0.744)	Beta	(23)
**PD**	0.72	(0.7, 0.735)	Beta	(23)

EoLC, end-of-life care; OS, overall survival; PD, progressive disease; PFS, progression-free survival.

### 2.5 Sensitivity analyses

Deterministic sensitivity analysis was used to see how changing the parameters affected the ICER. The parameter values were drawn from a 95% confidence interval or ±20% of the mean values, and the results are illustrated with a tornado diagram. In the probabilistic sensitivity analysis, we randomly sampled the input parameters and generated different costs, effectiveness, and ICER for each treatment strategy. An incremental cost-effectiveness scatterplot and cost-effectiveness acceptability curve were obtained from 10,000 Monte Carlo simulations.

## 3 Results

### 3.1 Base case results

We assessed the base-case results from the treatment effects, direct medical costs, and utility parameters. The cost and effectiveness results for atezo-bev and sorafenib are shown in **Table 2**. The total cost for each patient of the atezo-bev group was higher than that of sorafenib (atezo-bev: USD 180,348; sorafenib: USD 40,991). By applying a 3% annual discount rate, the atezo-bev group would cost USD 166,971, and sorafenib would cost USD 39,364. However, atezo-bev and sorafenib could increase patients’ survival time by 4.22 years and 1.45 years, respectively. After adjusting for the utility parameters, we obtained quality-adjusted life years in which the atezo-bev group was 3.06 years and sorafenib was 1.04 years. In addition, we discounted progression life years at 3% per year, which gives a quality-adjusted life expectancy of 2.68 years for the atezo-bev group and 0.98 years for the sorafenib group. Similar to sorafenib, the atezo-bev group had an incremental cost of USD 127,607 and incremental effectiveness of 1.7 quality-adjusted life years (QALYs). Thus, the ICER was USD 75,192 and the INMB was USD 29,609 per QALY. Based on the study, we found that ICER was less than WTP and INMB was greater than zero. This indicates that the atezo-bev group is a cost-effective treatment option in Taiwan.

**Table 2:** Base case results including costs and effectiveness.

Strategies	Cost		Effectiveness		
	Cost	Cost (disc.)	LYs	QALYs	QALYs (disc.)
**atezo-bev**	USD 180,348	USD 166,971	4.22	3.06	2.68
**Sorafenib**	USD 40,991	USD 39,364	1.45	1.04	0.98

disc., discounts (3%); LYs, life-years; QALYs, quality-adjusted life-years.

### 3.2 Sensitivity analyses

**Figure 2:**
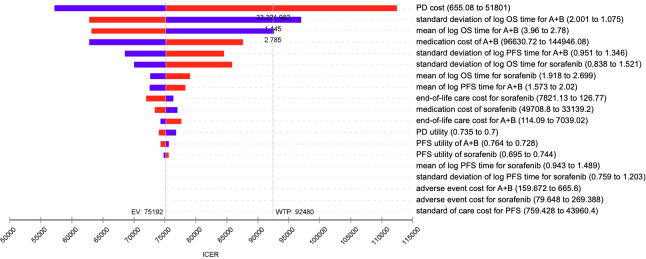
Tornado diagram – One-way sensitivity analysis of atezo-bev versus sorafenib. ICER, incremental cost-effectiveness ratio; WTP, willingness to pay; EV, expected value; PD, progressive disease; PFS, progression-free survival; OS, overall survival.

From the deterministic sensitivity analysis, the economic outcomes were significantly affected by the uncertainty of cost in the disease progression state, standard deviation, and mean of the log of overall survival time in the atezo-bev group. If the cost of disease progression was greater than USD 33,325, the standard deviation of log overall survival time in the lognormal distribution in the atezo-bev group was less than 1.145, or the mean value of log overall survival time in the lognormal distribution in the atezo-bev group was less than 2.785. The ICER might be greater than WTP, indicating that atezo-bev is not cost-effective. However, ICER did not appear to be affected by the uncertainty of the utility parameters (**Figure 2**).

**Figure 3:**
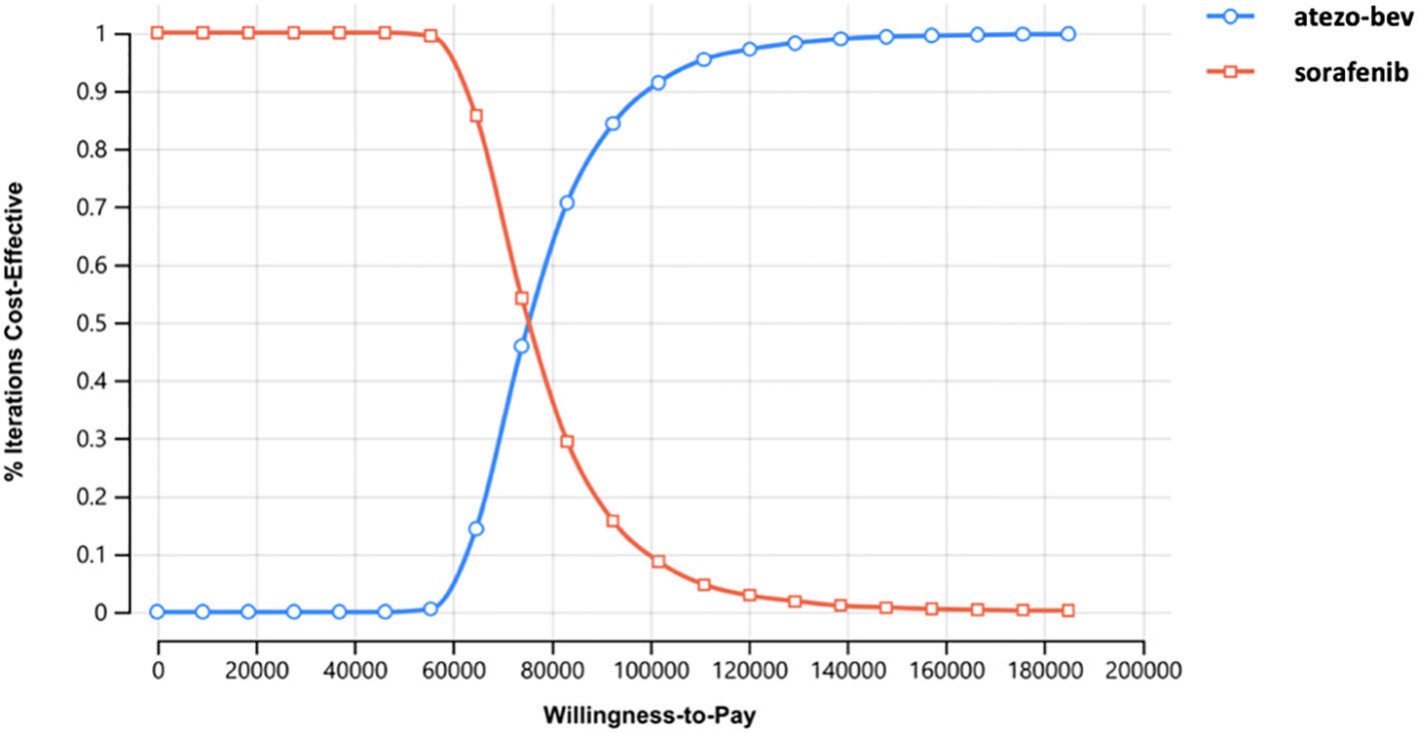
Cost-effectiveness acceptability curve of atezo-bev versus sorafenib.

According to the probabilistic sensitivity analysis, the incremental cost-effectiveness scatterplot showed that the ICER scatterplot was in the first quadrant, which means that most patients would gain effectiveness at a higher cost (**Appendix Figure 1**). The cost-effectiveness acceptability curves showed that the probability of atezo-bev being cost-effective compared with sorafenib was 84.31% and 15.69% at a WTP of USD 92,480 per QALY, respectively (**Figure 3**).

## 4 Discussion

This study indicates that atezo-bev is a cost-effective treatment strategy in Taiwan from the perspective of the NHIA. The base case result showed that the combination of atezo-bev improved survival by 1.7 QALYs with an incremental cost of USD 127,607 (ICER= USD 75,192/QALY). Our findings were robust in sensitivity analyses, especially probabilistic sensitivity analysis, which demonstrated that atezo-bev was 84.31% cost-effective. Economic simulations consistently showed that atezo-bev was the most cost-effective treatment approach.

The cost-effectiveness of atezo-bev may contribute to greater equity in healthcare access. Affordable and effective treatments are essential in ensuring that all HCC patients, regardless of their socioeconomic status, have access to the latest advancements in cancer care. This could help reduce health disparities within the HCC population.

### 4.1 Comparison with previous studies

In comparison with previous studies, it is evident that the cost-effectiveness of atezo-bev versus sorafenib varies based on the perspective of different countries and the economic evaluation models employed. Several studies, such as those by Su et al. and Zhang et al., evaluated this comparison from the perspective of the US payer (15, 16). Both studies employed a partitioned survival model and reported ICERs of USD 169,223/QALY and USD 322,500/QALY, respectively. Chiang et al. also approached this from the US payer perspective, utilizing a Markov model and reporting an ICER of USD 179,729/QALY (12).

From the perspective of China and the US, Wen et al. reported ICERs of $145,546.21 per QALY in China and $168,030.21 per QALY in the USA (13). In contrast, from the perspective of the Chinese healthcare system, Zhao et al. found that atezo-bev compared with sorafenib had an ICER of $27,630.63/QALY in China (17). Notably, our study reveals that the use of atezo-bev versus sorafenib in Taiwan results in a lower ICER per QALY gained compared to most of these previous studies.

To the best of our knowledge, this is the first economic evaluation of atezo-bev in Asia based on clinical trial results, specifically from a Chinese subpopulation, which included patients from mainland China, Hong Kong, and Taiwan (18). The significant effect on the Chinese subpopulation may explain why atezo-bev is more cost-effective in Taiwan than in other countries. While efficacy of atezo-bev in Chinese subpopulation is better than global population, the estimated incremental life expectancy in our study (1.7 QALYs) is longer than other studies (0.44–0.86 QALYs) (12, 13, 15–17). Recently, updated efficacy and safety data from IMbrave150 were released, and the results of subgroup analysis showed that the hazard ratio for death in the Asian region (excluding Japan) was 0.62 (0.42-0.93), compared to the rest of the world, which was 0.68 (0.50-0.93) (29). Atezo-bev and sorafenib had a median overall survival in the Asia (excluding Japan) of 22.8 months and 13.1 months, respectively. Updated efficacy data demonstrated that atezo-bev was superior to sorafenib in Asian countries.

We compared the cost parameters between the two countries based on the GDPs of Taiwan and the United States (**Appendix Table 1**). In Taiwan, the cost of atezo-bev was similar to that of the United States when weighted by GDP per capita. Regarding the medication cost of sorafenib, we observed that it was much cheaper in Taiwan than in the United States. As Taiwan has higher incremental costs than the United States, atezo-bev may not be cost-effective in Taiwan. However, previous studies showed that the ICER of atezo-bev versus sorafenib was USD 169,223/QALY from the United States payer perspective (15), and this study showed that the ICER in Taiwan was USD 75,192/QALY. One possible explanation is that Taiwan’s standard of care costs are much lower than those in the United States. According to the one-way sensitivity analysis results, the standard of care costs for disease progression might have the greatest impact on cost-effectiveness. Nevertheless, the standard care cost for disease progression in Taiwan is ten times cheaper than that in the United States. Atezo-bev is likely more cost-effective in Taiwan than in the United States because it has higher incremental effectiveness and lower incremental costs than the United States.

### 4.2 Implications of the study

In addition to considering clinical and methodological boundaries, the cost-effectiveness of atezo-bev should also be evaluated with regard to the complex socioeconomic conditions associated with the treatment of unresectable HCC. In light of the growing availability of effective cancer treatments, patients are often in the paradoxical position of having access to potentially life-saving medications but lacking the financial resources to afford them.

Budget constraints necessitate the rigorous evaluation of cancer immunotherapies by Taiwan’s National Health Insurance, potentially causing delays in patient access. Furthermore, the requirement for costly cancer therapies to undergo cost-effectiveness assessments for reimbursement in Taiwan, especially when their financial impact is projected to surpass NTD 500 million annually within the subsequent five years post-reimbursement, highlights the intricate balance between ensuring access to innovative treatments and managing the healthcare system’s financial sustainability.

Our study’s demonstration of atezo-bev’s cost-effectiveness compared to sorafenib, suggesting that certain innovative treatments can provide both clinical benefits and economic viability. Nevertheless, addressing the broader socioeconomic challenges in healthcare accessibility requires a sustained, collaborative effort among stakeholders, including policymakers, healthcare providers, and pharmaceutical companies.

### 4.3 Strengths and limitations

Although several studies have evaluated the cost-effectiveness of atezo-bev as a first-line treatment for HCC, most studies were considered from the US perspective. They evaluated the ICER from the IMbrave150 global study. In this study, we evaluated the economic outcomes of the Chinese subpopulation data on the effectiveness of atezo-bev as a first-line treatment to evaluate local effectiveness. Moreover, we conducted the AIC test and visual inspection and referred to real-world survival data of HCC to extrapolate the survival curves more accurately. We analyzed the NHIRD for real-world domestic costs, which could represent the direct medical expenses in Taiwan. In relation to the utilities assigned to progressive disease and progression-free survival, it’s crucial to highlight that our model incorporated an adjustment to account for the disutility associated with adverse events (AEs). Prior to this adjustment, the utility for atezo-bev in the progression-free state was 0.78, while for sorafenib in the same state, it was 0.77 (23). Meanwhile, the utility for the state of disease progression was 0.74 (23). However, post-adjustment to account for AE disutility, both the utility for sorafenib and the utility for disease progression were revised to 0.72, while the utility for atezo-bev was adjusted to 0.74 (23).

This adjustment reflects the impact of sorafenib’s side effects on patients’ quality of life. It suggests that the adverse effects associated with sorafenib treatment led to a reduction in the quality of life comparable to that experienced during disease progression. This observation underscores the clinical significance of the combination therapy (atezo-bev), emphasizing the importance of achieving optimal treatment outcomes with minimal adverse effects.

However, this study had some limitations. During the extrapolation part of the economic model, the long-term PFS and OS extrapolation are uncertain due to the limitation of clinical trial time. Without individual patient data from the IMbrave150 Chinese subpopulation study, we could not estimate the local utility value considering the Taiwanese tariff. Instead, we could only use the NICE utility parameters to evaluate quality-adjusted life months for patients with HCC. The tornado diagram shows that cost-effectiveness might be affected by the uncertainty associated with the overall survival of patients with atezo-bev. Currently, Taiwan has a limited number of patients receiving atezo-bev treatment, making it difficult to obtain real-world evidence regarding its long-term effectiveness.

Lenvatinib is another standard treatment for unresectable HCC in Taiwan, and the National Healthcare Insurance reimburses for this treatment. However, this analysis did not include lenvatinib, because the unavailability of published Kaplan- Meier curves that exclusively compared the Chinese subpopulation’s outcomes using lenvatinib or sorafenib. In other words, while the REFLECT study contained Chinese HCC patients, there was no accessible data that isolated the survival outcomes of only the Chinese patients in a head-to-head comparison between lenvatinib and sorafenib. This lack of directly comparative effectiveness data for the Chinese subpopulation posed a significant challenge in conducting the kind of indirect comparison required for our study. Furthermore, the study only showed that atezo-bev is a cost-effective treatment in Taiwan, and future research is needed to determine the budget impact of using atezo-bev in Taiwan.

## 5 Conclusion

From the perspective of NHIA in Taiwan, this study shows that atezo-bev is cost-effective as first-line treatment compared with sorafenib. While its reimbursement began on August 1, 2023, it is important to acknowledge that certain usage conditions and restrictions still exist, limiting early access for some patients. As a recommendation, we propose that the NHIA consider gradually expanding the scope of eligibility. In the future, the incorporation of real-world data into both effectiveness and economic evaluation can serve as a valuable foundation for Health Technology Reassessment initiatives.

## Data availability statement

The data analyzed in this study is subject to the following licenses/restrictions: The use of NHIRD is limited to research purposes only. Applicants must follow the Computer-Processed Personal Data Protection Law (http://www.winklerpartners.com/?p=987) and related regulations of National Health Insurance Administration and NHRI (National Health Research Institutes), and an agreement must be signed by the applicant and his/her supervisor upon application submission. All applications are reviewed for approval of data release. Requests to access these datasets should be directed to stcoollion@mohw.gov.tw.

## Ethics statement

The studies involving humans were approved by National Yang Ming Chiao Tung University (IRB No. YM110172E). The studies were conducted in accordance with the local legislation and institutional requirements. Written informed consent for participation was not required from the participants or the participants’ legal guardians/next of kin in accordance with the national legislation and institutional requirements.

## Author contributions

C-YT: Conceptualization, Data curation, Formal analysis, Methodology, Software, Writing – original draft. Y-WT: Methodology, Software, Supervision, Writing – review & editing. M-NS: Conceptualization, Methodology, Resources, Supervision, Writing – review & editing.
