# Supplementary material for: Cost-effectiveness analysis of atezolizumab plus bevacizumab versus sorafenib in first line treatment for Chinese subpopulation with unresectable hepatocellular carcinoma
Source: Front Oncol. 2023 Nov 8;13:1264417. doi: 10.3389/fonc.2023.1264417 (PMC10663301; doi:10.3389/fonc.2023.1264417)
Supplement: Supplementary file 1 [file Image_1.pdf]

Supplementary Material - Appendix Figure 1

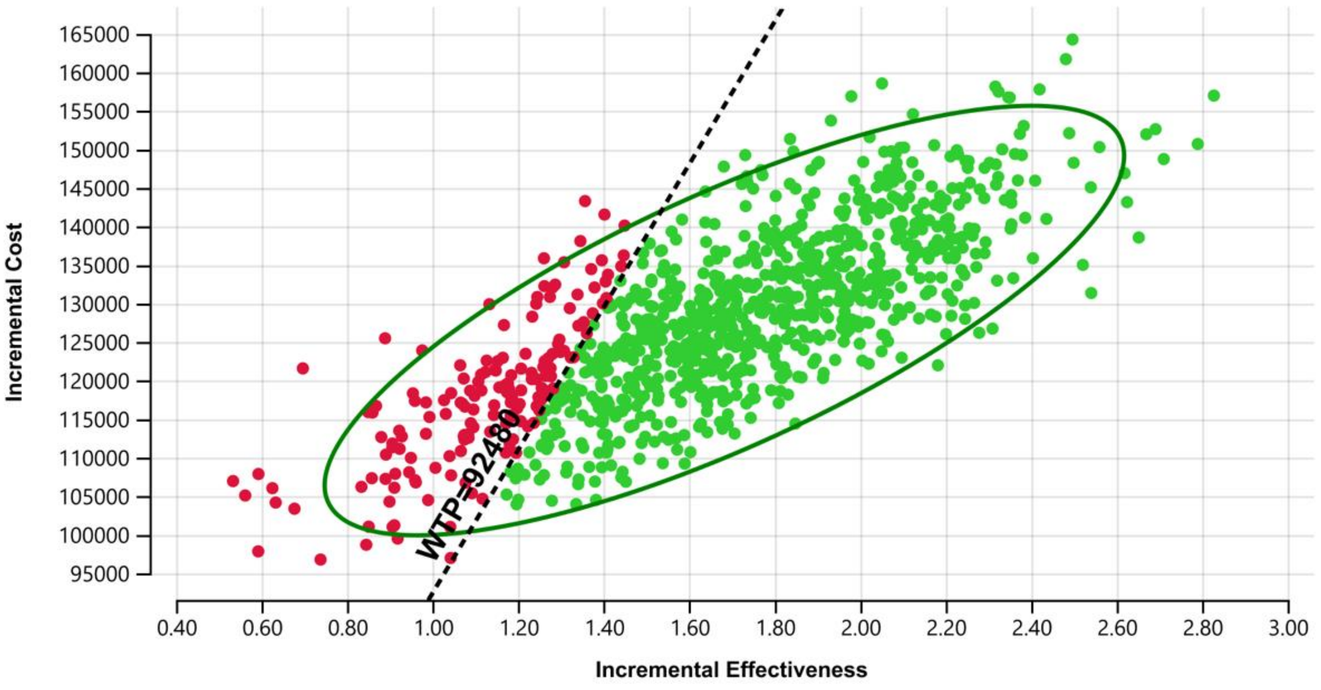


Appendix Figure 1: Incremental cost-effectiveness scatterplot of atezo-bev versus sorafenib. WTP, Willingness to pay
