# Supplementary material for: Cost-effectiveness analysis of atezolizumab plus bevacizumab versus sorafenib in first line treatment for Chinese subpopulation with unresectable hepatocellular carcinoma
Source: Front Oncol. 2023 Nov 8;13:1264417. doi: 10.3389/fonc.2023.1264417 (PMC10663301; doi:10.3389/fonc.2023.1264417)
Supplement: Supplementary file 2 [file Table_1.docx]

Supplementary Material - Appendix Table 1

Appendix Table 1: Comparison of costs parameters weighted by GDP per capita between Taiwan and United States

| USD  per month | atezo-bev | sorafenib | SOC (PD) | GDP per capita in 2020 | atezo-bev /GDP per capita | sorafenib/ GDP per capita | SOC (PD) / GDP per capita |
| --- | --- | --- | --- | --- | --- | --- | --- |
| Taiwan | USD 10,066 | USD 3,452 | USD 1,443 | USD 28,383 | 0.35 | 0.12 | 0.05 |
| US | USD 22,598 | USD 18,874 | USD 37,084 | USD 63,207 | 0.36 | 0.3 | 0.59 |
| atezo-bev, atezolizumab plus bevacizumab; GDP, gross domestic product; PD, progressive disease; SOC, standard of care; | | | | | | | |
